# Acute kidney disease and acute kidney injury biomarkers in coronary care unit patients

**DOI:** 10.1186/s12882-020-01872-z

**Published:** 2020-06-01

**Authors:** Yih-Ting Chen, Chang-Chyi Jenq, Cheng-Kai Hsu, Yi-Ching Yu, Chih-Hsiang Chang, Pei-Chun Fan, Heng-Chih Pan, I-Wen Wu, Wen-Jin Cherng, Yung-Chang Chen

**Affiliations:** 1 Department of Nephrology, Chang Gung Memorial Hospital, Keelung, Taiwan; 2 Kidney Research Center, Department of Nephrology, Chang Gung Memorial Hospital, Linkou, Taiwan; 3 Department of Neurology, Chang Gung Memorial Hospital, Keelung, Taiwan; 4 College of Medicine, Chang Gung University, Taoyuan, Taiwan; 5 Department of Cardiology, Chang Gung Memorial Hospital, Linkou, Taiwan; 6 Community Medicine Research Center, Chang Gung Memorial Hospital, Keelung, Taiwan; 7 Division of Nephrology, Department of Medicine, Keelung Chang Gung Memorial Hospital, No. 222, Maijin Rd., Anle Dist, Keelung City, Taiwan

**Keywords:** AKD, CCU, AKI, Biomarker and CKD

## Abstract

**Background:**

Acute kidney disease (AKD) describes acute or subacute damage and/or loss of kidney function for a duration of between 7 and 90 days after exposure to an acute kidney injury (AKI) initiating event. This study investigated the predictive ability of AKI biomarkers in predicting AKD in coronary care unit (CCU) patients.

**Methods:**

A total of 269 (mean age: 64 years; 202 (75%) men and 67 (25%) women) patients admitted to the CCU of a tertiary care teaching hospital from November 2009 to September 2014 were enrolled. Information considered necessary to evaluate 31 demographic, clinical and laboratory variables (including AKI biomarkers) was prospectively recorded on the first day of CCU admission for post hoc analysis as predictors of AKD. Blood and urinary samples of the enrolled patients were tested for neutrophil gelatinase-associated lipocalin (NGAL), cystatin C (CysC) and interleukin-18 (IL-18).

**Results:**

The overall hospital mortality rate was 4.8%. Of the 269 patients, 128 (47.6%) had AKD. Multivariate logistic regression analysis revealed that age, hemoglobin, ejection fraction and serum IL-18 were independent predictors of AKD. Cumulative survival rates at 5 years of follow-up after hospital discharge differed significantly (*p* < 0.001) between subgroups of patients diagnosed with AKD (stage 0A, 0C, 1, 2 and 3). The overall 5-year survival rate was 81.8% (220/269). Multivariate Cox proportional hazard analysis revealed that urine NGAL, body weight and hemoglobin level were independent risk factors for 5-year mortality.

**Conclusions:**

This investigation confirmed that AKI biomarkers can predict AKD in CCU patients. Age, hemoglobin, ejection fraction and serum IL-18 were independently associated with developing AKD in the CCU patients, and urine NGAL, body weight and hemoglobin level could predict 5-year survival in these patients.

## Background

Kidney damage lasting between 7 and 90 days after an acute kidney injury (AKI) is termed acute kidney disease (AKD) [1–3]. Although many studies have reported on AKI and chronic kidney disease (CKD), few studies have investigated AKD.

The causes of renal function impairment in patients admitted to a coronary care unit (CCU) are complex and multifactorial [4–7]. Cardio-renal syndrome (CRS) was first proposed by Ronco and colleagues in 2008 [8] in an attempt to clarify the crosstalk between the heart and kidneys. The CRS is defined as a pathophysiologic illness of the heart and kidneys in which acute or chronic dysfunction of one organ may induce acute or chronic dysfunction of the other. There are five categories in the CRS and the first, third and fifth types of CRS are associated with AKI. A recent study conducted in the CCU of Chang Gung Memorial Hospital reported an incidence of AKD of 29% [9]. The use of AKI biomarkers in the diagnosis and prognosis of AKI in critically ill patients has also been demonstrated [10, 11]. However, the role of AKI biomarkers in the diagnosis of AKD and the impact of AKD on mortality are unclear, and new classification systems for AKD may improve standardization of the diagnosis and staging of this clinical syndrome.

Accordingly, there were two aims in this study. First, to explore the predictors of AKD in CCU patients and identify associations between AKI renal biomarkers and patient prognosis. Second, to analyze the severity of AKD as a clinical predictor of long-term adverse outcomes.

## Methods

### Study design and patient population

**Fig. 1:**
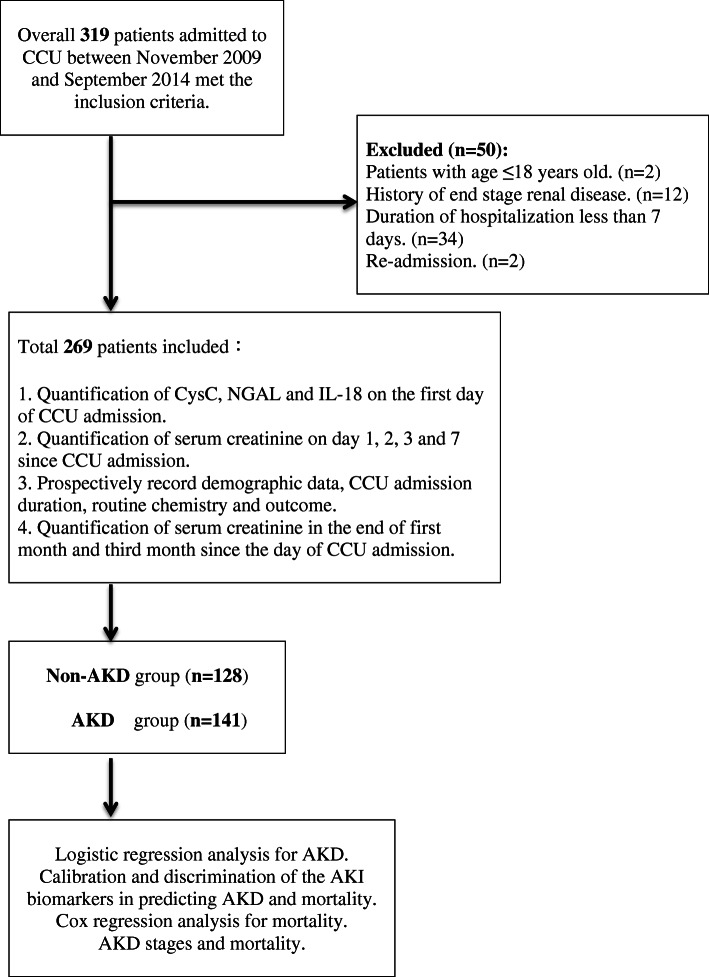
Study flow chart. AKI, acute kidney injury; AKD, acute kidney disease; CCU, coronary care unit; IL-18, interleukin 18; NGAL, neutrophil gelatinase-associated lipocalin; CysC, cystatin C

This clinical research was conducted in the CCU of one tertiary care teaching hospital in Taiwan between November 2009 and September 2014. The Institutional Review Board at Chang Gung Memorial Hospital approved the study protocol (approval No. 201702274B0). Patients who exhibited any of the comorbidities associated with AKI and AKD were enrolled in this investigation. The following risk factors were considered to be associated with AKI and AKD: age > 65 years, diabetes mellitus, congestive heart failure (functional class III or IV), and chronic kidney disease (CKD, defined as an estimated glomerular filtration rate ≤ 60 mL/min). All patients provided written informed consent to join the study. Overall, 319 patients were included initially. Patients aged ≤18 years (*n* = 2), those with end-stage kidney disease (*n* = 12), duration of hospital stay < 7 days (*n* = 34) and readmission (*n* = 2) were excluded. In total, 269 critically ill patients were enrolled (Fig. 1).

### Data collection

Post hoc analysis of a prospectively collected database was used to examine the following variables during CCU admission: demographic characteristics, primary diagnosis, routine biochemistry tests and serial serum creatinine on day 1, 2, 3 and 7. Serum creatinine levels at the end of first month and third month after CCU admission were collected via record reviews. Levels of interleukin-18 (IL-18), cystatin C (CysC), and neutrophil gelatinase-associated lipocalin (NGAL) on the first day of CCU admission were measured in urine and serum samples from all of the enrolled cases. The primary study endpoint was the diagnosis of AKD in order to determine the predictive value of AKI biomarkers in the diagnosis of AKD. The secondary outcome was 5-year mortality to assess the prognostic value of the biomarkers. After hospital discharge, 5 years of follow-up data were evaluated via medical records or telephone interviews as needed.

### Definitions

Acute myocardial injury was defined according to the Third Universal Definition of Myocardial Infarction, ESC/ACCF/AHA/WHF Expert Consensus Document [12] as: an increase and/or decrease in cardiac biomarkers (we evaluated troponin I in this study) >99th percentile of the upper reference limit, plus one or more of the following: 1. Development of pathological Q waves. 2. Imaging evidence of the new loss of cardiac muscles or new regional wall motion abnormalities. 3. Symptoms of myocardial ischemia. 4. New left bundle branch block or significant new changes in ST-segment–T wave (ST–T). 5. Intracoronary thrombus. Heart failure was diagnosed according to the Framingham criteria [13], and the American College of Chest Physicians/Society of Critical Care Medicine (ACCP/SCCM) Consensus Conference criteria were used to define respiratory failure and sepsis [14] as the the need for mechanical ventilation and a systemic response to infection, respectively. AKI was defined according to the clinical practice guidelines of the Kidney Disease: Improving Global Outcomes (KDIGO) AKI work group [15] as: an increase in serum creatinine levels of ≥0.3 mg/dL within 48 h; an increase of ≥1.5 times the baseline value within the last 7 days; or urine output < 0.5 mL/kg/h for 6 h. The Chinese Modification of Diet in Renal Disease equation was used to calculate the estimated glomerular filtration rate [16]. The stage of AKD staging was determined using baseline serum creatinine levels, which were measured during the first CCU admission.

**Fig. 2:**
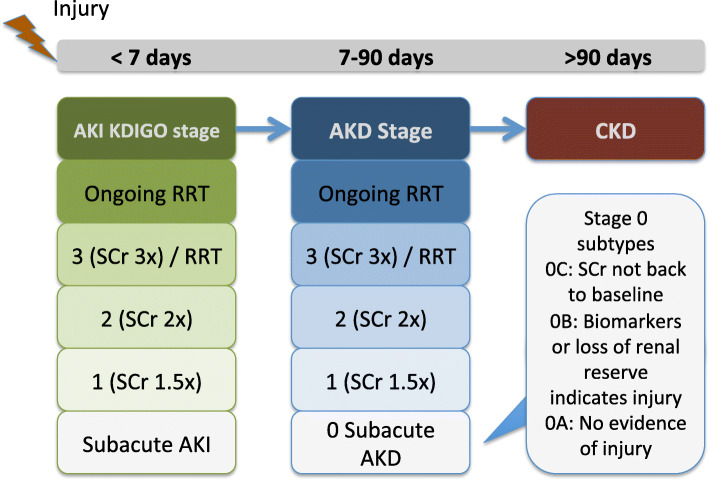
Acute kidney injury and acute kidney disease stages. AKI, acute kidney injury; AKD, acute kidney disease; CKD, chronic kidney disease; RRT, renal replacement therapy; KDIGO, Kidney Disease Improving Global Outcomes; SCr, serum creatinine

AKD and renal recovery were defined according to the consensus report of the Acute Disease Quality Initiative (ADQI) 16 Workgroup and the staging criteria for AKD in San Diego, USA on November 8–10, 2015 [1]. Patients were grouped according to AKD stage as 0A, 0B, 0C, 1, 2 and 3. The stages of AKD were defined as follows: stage 0, patients with incomplete recovery from AKI; stage 0A, patients without damage markers or structural deficits after an AKI event who are still at risk of long-term events; stage 0B, patients with ongoing kidney injury, damage, or loss of renal functional reserve even though the serum creatinine level has returned to baseline levels; stage 0C, patients with a serum creatinine level higher than but within 1.5 times of the baseline level; stage 2, patients with a serum creatinine level 2.0–2.9 times higher than the baseline level; and stage 3, patients with a serum creatinine level > 3.0 times higher than baseline or ≥ 4.0 mg/dl, or an ongoing need for renal replacement therapy (Fig. 2). No patient met the criteria for stage 0B due to a lack of biomarker data during the follow-up period from day 7 to day 90.

### Sampling and quantifying urinary and serum biomarkers

Blood and urine samples were collected in sterile non-heparinized tubes immediately after CCU admission, centrifuged at 1500 rpm for 10 min. The samples were then stored at − 80 °C until further analysis. Levels of CysC and NGAL in urine and serum were measured in duplicate using a single enzyme-linked immunosorbent assay (R&D Systems, Minneapolis, MN, USA), and IL-18 levels were measured using another single a single enzyme-linked immunosorbent assay (Medical and Biologic Laboratories, Nagoya, Japan) according to the manufacturers’ instructions. Serum levels of B-type natriuretic peptide were measured using a commercial immunoassay, and an autoanalyzer was used to measure levels of high-sensitive C-reactive protein.

### Statistical analysis

Continuous variables are presented as mean ± standard error unless otherwise stated. The patients with and without AKD were compared in the primary analysis, and the distribution of each variable was evaluated using the Kolmogorov-Smirnov test. The means of continuous and normally distributed variables were compared using the Student’s *t*-test, and the Mann-Whitney *U* test was used for non-normally distributed variables. The chi-square test or Fisher’s exact test was used to compare categorical variables. The chi-square test for trends was used to analyze categorical data associated with the stage of AKD. Univariate analysis was used to identify the predictors of AKD. The statistically significant (*p* < 0.001) variables in the univariate analysis were then entered into multivariate logistic regression analysis using backward stepwise selection. Univariate Cox proportional hazard analysis was used to identify the statistically significant (*p* < 0.05) risk factors for 5-year mortality. Multivariate Cox analysis was then used with these risk factors to identify the independent predictors of 5-year survival.

The Hosmer-Lemeshow goodness-of-fit test was used to assess calibration of the number of predicted and actual deaths for all probabilities. The area under a receiver operating characteristic curve was used to assess discrimination, and compared using a nonparametric approach.

The Kaplan-Meier method was used to plot cumulative survival curves, which were compared using the log-rank test. All statistical tests were two-tailed, and a *p* value of < 0.05 was considered to be statistically significant.

## Results

### Study population characteristics

Overall, 269 patients (202 men and 67 women) with a mean age of 64 years were investigated. AKD was diagnosed in 128 patients (47.6%) and 13 patients (4.8%) died during hospitalization. The patients in the AKD group had a significantly higher incidence of receiving renal replacement therapy during CCU admission (5.5% vs. 0.7%; *p* = 0.029), a 2.5-fold higher rate of in-hospital mortality (7.1% vs. 2.8%; *p* = 0.154), and a higher 5-year all-cause mortality rate (22.7% vs. 14.2%; *p* = 0.083).

### AKD and AKI biomarkers

Table 1 lists the demographic data and clinical characteristics of the AKD and non-AKD groups. The AKD group was older, had a higher incidence rate of sepsis, higher body temperature, and higher respiratory rate than the non-AKD group. In addition, the AKI group had higher levels of AKI biomarkers including serum IL-18, NGAL, and CysC, and urine NGAL. There were also significant differences in serum high-sensitive C-reactive protein, B-type natriuretic peptide, albumin and left ventricular ejection fraction measured by echocardiography between the two groups (Table 1). The unit conversion table is provided in Additional file 1: Supplementary Table 1.

**Table 1:** Baseline demographic and clinical data in the patients with and without AKD

	All Patients (***n*** = 269)	AKD (***n*** = 128)	Non-AKD (***n*** = 141)	***p***-value
**Demographic and clinical variables**				
Age (years)	64 ± 1	68 ± 1	61 ± 1	< 0.001
Gender, Male(%)	202(75)	97(76)	105(74)	NS(0.888)
Body weight (kg)	66.3 ± 0.8	65.2 ± 1.1	67.4 ± 1.0	NS(0.157)
Diabetes mellitus, *n*(%)	110(41)	58(45)	52(37)	NS(0.173)
Hypertension, *n*(%)	163(61)	85(66)	78(55)	NS(0.08)
Sepsis, *n*(%)	15(5.6)	15(12)	0(0)	0.001
Body temperature (°C)	36.9 ± 0.1	37.1 ± 0.1	36.8 ± 0.1	0.026
Respiratory rate(/min)	22 ± 0	23 ± 1	21 ± 0	< 0.001
Mean arterial pressure (mmHg)	90 ± 3	92 ± 7	89 ± 1	NS(0.608)
Blood sugar (mg/dl)	160 ± 5	166 ± 9	155 ± 6	NS(0.288)
AKI biomarkers				
Serum IL-18 (ng/ml)	350 ± 20	454 ± 36	256 ± 15	< 0.001
Urine IL-18 (ng/ml)	61 ± 2	64 ± 3	59 ± 2	NS(0.165)
Serum NGAL (ng/ml)	112 ± 9	138 ± 14	88 ± 10	0.004
Urine NGAL (ng/ml)	46 ± 10	77 ± 20	20 ± 4	0.007
Serum CysC (mg/L)	1.9 ± 0.1	2.4 ± 0.2	1.5 ± 0.1	0.001
Urine CysC (mg/L)	1.0 ± 0.5	1.8 ± 1.1	0.4 ± 0.2	NS(0.213)
**Other Laboratory examinations**				
Serum creatinine baseline (mg/dL)	1.36 ± 0.07	1.44 ± 0.09	1.15 ± 0.10	0.029
Serum creatinine 0–24 h (mg/dL)	1.64 ± 0.13	1.96 ± 0.18	1.07 ± 0.14	< 0.001
Serum creatinine 24–48 h (mg/dL)	1.50 ± 0.13	1.81 ± 0.18	0.93 ± 0.07	< 0.001
Serum creatinine 48–72 h (mg/dL)	1.49 ± 0.11	1.79 ± 0.16	1.02 ± 0.12	< 0.001
Serum creatinine 7-day (mg/dL)	1.87 ± 0.20	1.96 ± 0.21	0.89 ± 0.13	< 0.001
Serum creatinine 1-month (mg/dL)	1.60 ± 0.13	1.68 ± 0.15	1.28 ± 0.25	NS(0.208)
Serum creatinine 3-month (mg/dL)	1.59 ± 0.14	1.64 ± 0.16	1.31 ± 0.31	NS(0.397)
Hemoglobin (g/dL)	12.9 ± 0.15	12.0 ± 0.2	13.7 ± 0.2	< 0.001
Leukocytes (×10^3^/μL)	9.5 ± 0.3	10.0 ± 0.6	9.1 ± 0.3	NS(0.174)
Serum sodium (mmol/L)	139 ± 0	139 ± 0	139 ± 0	NS(0.379)
hsCRP (mg/L)	31 ± 3	42 ± 6	20 ± 3	0.001
Troponin I (ng/ml)	6.6 ± 1.0	5.4 ± 1.2	7.7 ± 1.7	NS(0.272)
BNP (pg/ml)	725 ± 70	1025 ± 106	382 ± 71	< 0.001
Ejection fraction (%)	54 ± 1	51 ± 2	57 ± 1	0.004
Albumin (g/L)	3.7 ± 0.0	3.5 ± 0.1	3.9 ± 0.0	< 0.001

*AKI* Acute kidney injury, *IL-18* Interleukin 18, *NGAL* Neutrophil gelatinase-associated lipocalin, *CysC* Cystatin C, *hsCRP* High sensitivity C-reactive protein, *BNP* B-type natriuretic peptide, *NS* Not significant

Univariate analysis identified that 15 of the 31 variables (Tables 1 and 2) were associated with AKD. Multivariate analysis identified hemoglobin, age, ejection fraction and serum IL-18 as independent predictors of AKD (Table 2). A sensitivity analysis in which patients with CKD were excluded is provided in Additional file 2: Supplementary Table 2.

**Table 2:** Logistic regression analysis for AKD according to baseline prognostic factors

Parameter	Beta Coefficient	Standard error	Odds ratio (95% CI)	***p***-value
**Univariable logistic regression**				
Age	0.037	0.010	1.038(1.018–1.057)	< 0.001
Hemoglobin	−0.321	0.059	0.725(0.646–0.814)	< 0.001
Body temperature	0.327	0.149	1.387(1.036–1.858)	0.028
Respiratory rate	0.077	0.022	1.080(1.034–1.128)	0.001
Serum creatinine 0–24 h	0.821	0.283	2.272(1.306–3.954)	0.004
Serum creatinine 24–48 h	1.605	0.561	4.980(1.660–14.940)	0.004
Serum creatinine 48–72 h	0.774	0.261	2.168(1.301–3.614)	0.003
hsCRP	0.014	0.004	1.014(1.005–1.023)	0.002
Ejection fraction	−0.022	0.008	0.978(0.963–0.993)	0.004
BNP	0.001	0.000	1.001(1.001–1.002)	< 0.001
Albumin	−1.581	0.342	0.206(0.105–0.402)	< 0.001
Serum IL-18	0.003	0.001	1.003(1.002–1.005)	< 0.001
Serum NGAL	0.003	0.001	1.003(1.001–1.006)	0.009
Urine NGAL	0.006	0.003	1.007(1.001–1.012)	0.011
Serum CysC	0.000	0.000	1.000(1.000–1.001)	0.001
**Multivariable logistic regression**				
Hemoglobin	−0.241	0.085	0.786(0.665–0.928)	0.004
Age	0.034	0.014	1.034(1.007–1.063)	0.014
Ejection fraction	−0.025	0.011	0.975(0.955–0.996)	0.021
Serum IL-18	0.002	0.001	1.002(1.000–1.004)	0.015

*IL-18* Interleukin 18, *NGAL* Neutrophil gelatinase-associated lipocalin, *CysC* Cystatin C, *hsCRP* High sensitivity C-reactive protein, *BNP* B-type natriuretic peptide

Goodness-of-fit indices from Hosmer-Lemeshow chi-square analysis were used to assess calibration of the predicted risk of AKD and the predictive accuracy of serum levels of CysC and IL-18 and both serum and urine levels of NGAL (Table 3). The discriminatory power of these AKI biomarkers are also shown in Table 3.

**Table 3:** Calibration and discrimination of the AKI biomarkers in predicting AKD and 5-year mortality

	Calibration			Discrimination		
	Goodness –of-fit (χ2)	df	*p-value*	AUROC±SE	95%CI	*p*-value
***For AKD prediction***						
Serum IL-18	10.518	8	0.231	0.698 ± 0.033	0.634–0.762	< 0.001
Serum NGAL	5.756	8	0.675	0.630 ± 0.034	0.563–0.697	< 0.001
Urine NGAL	12.980	8	0.113	0.660 ± 0.036	0.591–0.730	< 0.001
Serum CysC	9.448	8	0.306	0.663 ± 0.033	0.598–0.728	< 0.001
***For 5-year mortality prediction***						
Serum IL-18	0.712	8	0.999	0.522 ± 0.048	0.429–0.616	0.635
Serum NGAL	9.717	8	0.285	0.573 ± 0.047	0.482–0.665	0.113
Urine NGAL	19.266	8	0.014	0.712 ± 0.047	0.620–0.803	< 0.001
Serum CysC	7.780	8	0.455	0.554 ± 0.048	0.460–0.648	0.241

No statistically significant AUROC differences between serum IL-18, serum NGAL, urine NGAL and serum CysC for AKD prediction*.

Statistically significant AUROC differences between urine NGAL vs. serum IL-18, serum NGAL and serum CysC for 5-year mortality prediction, all *p*-values < 0.01*.

*AKI* Acute kidney injury, *AKD* Acute kidney disease, *IL-18* Interleukin 18, *NGAL* Neutrophil gelatinase-associated lipocalin, CysC Cystatin C, *AUROC* Area under the receiver operating characteristic curve

*Pairwise comparisons of ROC curves, by Delong method, 1988

### Five-year all-cause mortality of the AKD patients and AKI biomarkers

Cox proportional hazard analysis identified 11 variables with prognostic value for 5-year all-cause mortality (Table 4). Multiple Cox regression analysis showed that urine NGAL, body weight and hemoglobin on first day of CCU admission were independent risk factors for 5-year all-cause mortality.

**Table 4:** Cox regression analysis for all-cause 5-year mortality

Parameter	Beta coefficient	Standard error	Hazard ratio(95% CI)	*p-*value
***Univariate hazard analysis***				
Hemoglobin	−0.207	0.057	0.813(0.726–0.909)	< 0.001
Age	0.023	0.011	1.024(1.001–1.046)	0.037
Gender, Male	−0.705	0.300	0.494(0.274–0.890)	0.019
Body weight	−0.044	0.014	0.957(0.932–0.983)	0.001
Serum CysC	0.000	0.000	1.000(1.000–1.000)	0.015
Urine NGAL	0.002	0.001	1.002(1.001–1.003)	0.001
Urine CysC	0.000	0.000	1.000(1.000–1.000)	< 0.001
hsCRP	0.006	0.003	1.006(1.001–1.011)	0.029
Albumin	−1.046	0.261	0.351(0.211–0.586)	< 0.001
Serum sodium	−0.117	0.042	0.890(0.820–0.966)	0.005
BNP	0.001	0.000	1.001(1.000–1.001)	< 0.001
***Multivariate hazard analysis***				
Urine NGAL	0.003	0.001	1.003(1.001–1.005)	0.006
Body weight	−0.040	0.018	0.961(0.927–0.996)	0.03
Hemoglobin	−0.268	0.104	0.765(0.625–0.937)	0.01

*NGAL* Neutrophil gelatinase-associated lipocalin, *CysC* Cystatin C, *hsCRP* High sensitivity C-reactive protein, *BNP* B-type natriuretic peptide

**Fig. 3:**
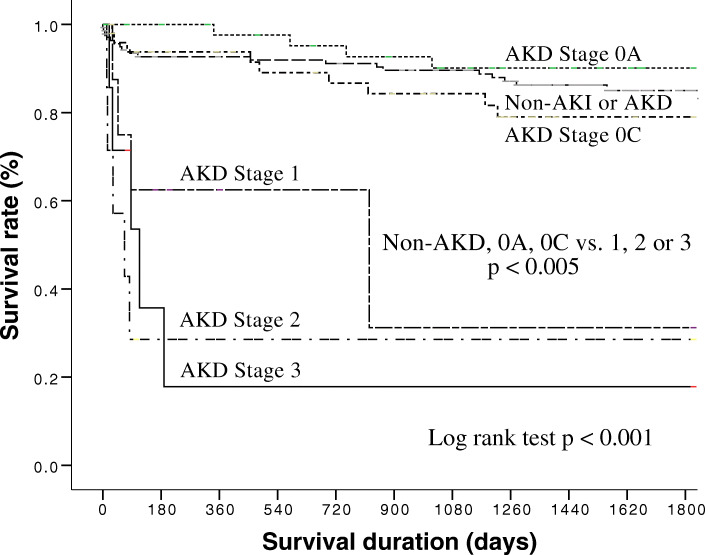
Cumulative survival rates for the 269 patients by stage of AKD. AKD, acute kidney disease; CCU, coronary care unit

**Fig. 4:**
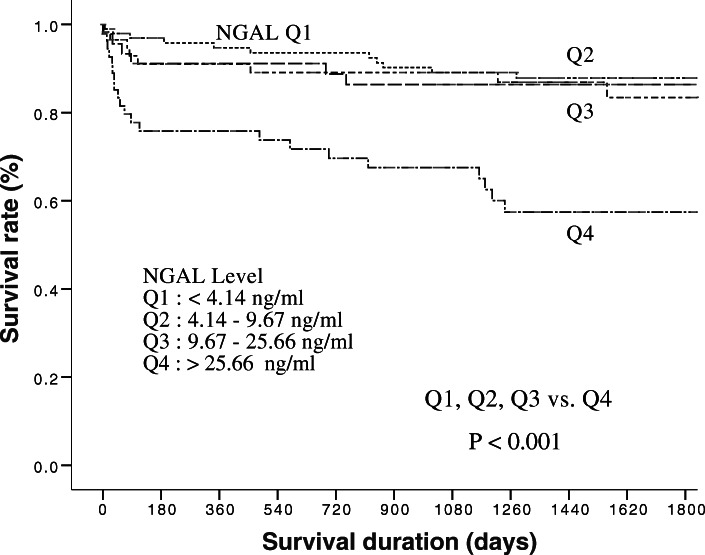
Cumulative survival rates for the 269 patients by urine NGAL quartile on the first day of CCU admission. AKD, acute kidney disease; CCU, coronary care unit; NGAL, neutrophil gelatinase-associated lipocalin

The 5-year all-cause mortality rates were 14.2% (20/141) for the non-AKD group, 10.2% (5/49) for AKD stage 0A, 17.3% (9/52) for AKD stage 0C, 41.7% (5/12) for AKD stage 1, 62.5% (5/8) for AKD stage 2, and 71.4% (5/7) for AKD stage 3 (chi-square for trend; *p* < 0.001). The odds ratios for AKD stage were 0.688 for AKD stage 0A versus non-AKD (*p* = 0.480), 1.266 for stage 0C versus non-AKD (*p* = 0.591), 4.321 for stage 1 versus non-AKD (*p* = 0.021), 10.083 for stage 2 versus non-AKD (*p* = 0.003), and 15.125 for stage 3 versus non-AKD (*p* = 0.002) (Table 5). Cumulative survival rates differed significantly (*p* < 0.005) between group 1 (non-AKI or AKD, AKD stage 0A and 0C) and group 2 (AKD stage 1, 2 and 3) (Fig. 3). In addition, the 5-year survival rates also differed significantly between the fourth quarter of urine NGAL (Q4: > 25.66 ng/ml) and the other three quarters (Q1: < 4.14 ng/ml; Q2: 4.14–9.67 ng/ml; and Q3: 9.67–25.66 ng/ml) (Fig. 4).

**Table 5:** AKD stages and 5-year all-cause-mortality

AKD stage	n	5-year all-cause-mortality (%)	Beta-coefficient	Standard error	Odds ratio, 95% CI	***p-***value
Non-AKD	141	14.2	–	–	1 (reference)	–
0A	49	10.2	−0.375	0.530	0.688 (0.243–1.943)	NS (0.480)
0C	52	17.3	0.236	0.439	1.266 (0.536–2.993)	NS (0.591)
1	12	41.7	1.464	0.633	4.321 (1.249–14.953)	0.021
2	8	62.5	2.311	0.769	10.083 (2.233–45.531)	0.003
3	7	71.4	2.716	0.871	15.125 (2.745–83.350)	0.002

*AKD* Acute kidney disease

## Discussion

The consensus report of the ADQI 16 Workgroup proposed the term AKD to define the pathway of renal pathophysiological processes that are continuing after AKI [1]. In this study, we found that clinical and laboratory variables (including AKI biomarkers) obtained during the first day of CCU admission were of predictive value for future AKD. Most CCU patients are diagnosed with coronary artery disease and related acute myocardial infarction. Peri-coronary artery catheterization-related contrast-associated AKI, post-acute myocardial infarction decompensated heart failure, hypoxemic respiratory failure and preexisting CKD have all been reported to contribute to the deterioration of renal function and to increase in-hospital mortality [17, 18]. In addition, ongoing heart failure, factors related to prolonged renal recovery (age, underlying diseases such as diabetes mellitus or hypertension, CKD or not, chronic medication usage) and intrinsic renal parenchymal injury are all related to the development of future AKD.

Previous studies have reported that AKI biomarkers including CysC, IL-18 and NGAL can improve the diagnosis of intrinsic AKI in CCU patients [9, 10]. In this study, we identified that age, ejection fraction, levels of hemoglobin and serum IL-18 were independent predictors of AKD in our CCU patients. Type 1 CRS indicates acute cardiac dysfunction leading to acute kidney dysfunction [19]. The proposed mechanisms include decreased cardiac output and renal perfusion pressure, venous congestion, neurohormonal dysregulation and systemic inflammation. Decreased ejection fraction reflects decreased cardiac output and renal hypoperfusion, which in turn causes functional AKI and parenchymal damage. Delayed improvement in renal function and prolonged renal parenchymal injury may lead to AKD. Previous studies have reported associations between serum IL-18 and a diagnosis of AKI, cardiac and renal outcomes. Striz et al. reported a higher level of serum IL-18 in patients with acute renal allograft rejection compared to those with an uncomplicated transplantation, and also that IL-18 mRNA was released in response to increased IFN-γ and TNF-α production [20]. In addition, a Japanese study reported that serum IL-18 was correlated with carotid intima-media thickness and urinary albumin excretion in diabetic nephropathy patients [21]. Therefore, serum IL-18 level may be a predictor of both cardiovascular diseases and renal outcome in patients with type 2 diabetes mellitus. Moreover, a study of middle-aged Europeans also reported that serum IL-18 level was an independent predictor of coronary events [22]. Furthermore, IL-18 genetic variations have been reported to influence the serum levels of IL-18 and the clinical outcomes of patients with coronary artery disease [23]. In the present study, the higher level of serum IL-18 may reflect more severe renal parenchymal injury, meaning more extensive tissue damage which may have prolonged the recovery time from AKI. AKD is diagnosed when serum creatinine fails to return to baseline or there is biomarker evidence of decreased renal reserve after 7 days.

The levels of AKI biomarkers have been reported to be independent predictors of short-term and long-term mortality [9, 24]. Urine NGAL has been shown to predict renal outcomes (kidney intrinsic injury, AKI progression, renal replacement therapy) and clinical outcomes in children undergoing cardiac surgery, critically ill intensive care unit patients, septic AKI patients and patients who visited emergency rooms [4, 25–28]. In addition, urine NGAL was shown to predict 28-day mortality in 109 intensive care unit patients in a study by Kümpers et al. [29]. However, very few studies have investigated associations between AKI biomarkers and long-term clinical prognosis. In the present study, we demonstrated that urine NGAL could independently predict 5-year mortality in our CCU patients, especially when the urine NGAL level exceeded 25.66 ng/ml.

In addition, anemia is a known predictor of re-hospitalization and mortality in patients with congestive heart failure [30, 31]. Cardiac renal anemia syndrome, characterized by concomitant cardiorenal dysfunction and anemia, highlights the importance of anemia in these patients [32]. The presence of anemia can increase the impact of cardiac or renal dysfunction on morbidity and mortality. The SENIOR study reported that the 309 included anemic patients (10%) were older and had poorer renal function (*p* < 0.05) compared to those without anemia. In addition, anemia has been reported to be an independent risk factor for all-cause mortality and hospitalization for cardiovascular disease [33]. In the present study, we demonstrated that body weight and hemoglobin and urine NGAL levels were independently associated with the risk of all-cause 5-year mortality in our CCU patients, which is consistent with previously published studies.

Furthermore, we observed that a higher AKD stage was correlated with higher 5-year all-cause mortality (patients with AKD stage 3 had a 15-fold higher risk of mortality compared to those without AKD). To the best of our knowledge, this is the first study to report an association between the severity of AKD and 5-year clinical outcomes in CCU patients. Interestingly, we observed that the 5-year cumulative survival rate of the patients diagnosed with AKD stage 0A (normalized creatinine level post-AKI) was slightly higher than in the patients without AKI and in those with AKD alone, despite being statistically insignificant. A previous meta-analysis demonstrated that patients who were referred to a nephrologist earlier had reduced mortality and complications of CKD [34]. We advised most of our patients who presented with AKD to receive follow-up with a nephrologist after being discharged from the CCU to monitor their health condition, including important risk factors. This kind of surveillance may help to lower the 5-year mortality rate of patients with AKD stage 0A.

### Study limitations

There are several limitations to the present study. First, it was conducted at one single center, and most of the patients were of East Asian ethnicity. Therefore, extrapolation of our results to other patient groups needs to be validated. Second, this study involved prospective data collection and post hoc analysis, so AKI biomarker data (including IL-18, serum and urine CysC) during the observation interval of AKD (i.e., 7 days to 3 months) were lacking, and therefore we could not accurately define the number of patients with AKD stage 0B. Third, only single measurements of AKI biomarkers were collected, so we could not observe continuous time-course changes in the AKI biomarkers. Fourth, there are inherent limitations with regards to the predictive accuracy of logistic regression analysis. Fifth, uric acid levels were not measured, and so we could not investigate whether hyperuricemia contributes to AKI and AKD, or whether it is simply a consequence of a decline in glomerular filtration rate and relative hypovolemia. Sixth, community-acquired AKI may have been mis-diagnosed as CKD as we used first serum creatinine data during admission, and the true incidence of AKI may have been underestimated. Finally, considering the intensive care unit setting and observational design, further prospective randomized controlled trials are necessary to validate the cost-efficacy of using AKI biomarkers to improve clinical outcomes.

## Conclusions

In conclusion, this investigation confirmed that AKI biomarkers can be used to predict AKD in CCU patients. Age, ejection fraction, hemoglobin and serum IL-18 were independently associated with the CCU patients who developed AKD. In addition, body weight, hemoglobin and urine NGAL could predict 5-year survival in the patients who were admitted to the CCU and diagnosed with AKD.

## Supplementary information

**Additional file 1 Supplementary Table 1.** The unit conversion table.

**Additional file 2 Supplementary Table 2.** Logistic regression analysis for AKD according to baseline prognostic factors after excluding patients with CKD.

## Data Availability

The datasets generated and/or analyzed during the current study are not publicly available due the specification of the Institutional Review Board at Chang Gung Memorial Hospital but are available from the corresponding author on reasonable request.

## Abbreviations

ADQI: Acute Disease Quality Initiative
AKI: Acute kidney injury
AKD: Acute kidney disease
CCU: Coronary care unit
CKD: Chronic kidney disease
CRS: Cardio-renal syndrome
IL-18: Interleukin 18
NGAL: Neutrophil gelatinase-associated lipocalin
CysC: Cystatin C
