# Supplementary material for: Acute kidney disease and acute kidney injury biomarkers in coronary care unit patients
Source: BMC Nephrol. 2020 Jun 1;21:207. doi: 10.1186/s12882-020-01872-z (PMC7268535; doi:10.1186/s12882-020-01872-z)
Supplement: Supplementary file 1 — Additional file 1 Supplementary Table 1. The unit conversion table. [file 12882_2020_1872_MOESM1_ESM.docx]

Additional file 1.

Supplementary Table 1. The unit conversion table

| **Parameter** | **Reference Range, Conventional Unit** | **Conventional Unit** | **Conversion Factor (Multiply by)** | **Reference Range, SI Unit** | **SI Unit** |
| --- | --- | --- | --- | --- | --- |
| Serum creatinine | Male: 0.64-1.27, Female: 0.44-1.03 | mg/dL | 88.4 | Male: 56.6-112.3, Female: 38.9-91.1 | μmol/L |
| Blood sugar | 70-100 | mg/dL | 0.0555 | 3.9-5.6 | mmol/L |
| High sensitivity C-reactive protein | 0.08-3.1 | mg/L | 9.524 | 0.76-29.5 | nmol/L |
| Troponin I | <0.3 | ng/mL | 1.0 | <0.3 | μg/L |
| Albumin | 3.5-5.5 | g/dL | 10 | 35-55 | g/L |
