# Supplementary material for: Acute kidney disease and acute kidney injury biomarkers in coronary care unit patients
Source: BMC Nephrol. 2020 Jun 1;21:207. doi: 10.1186/s12882-020-01872-z (PMC7268535; doi:10.1186/s12882-020-01872-z)
Supplement: Supplementary file 2 — Additional file 2 Supplementary Table 2. Logistic regression analysis for AKD according to baseline prognostic factors after excluding patients with CKD. [file 12882_2020_1872_MOESM2_ESM.docx]

Additional file 2.

Supplementary Table 2. Logistic regression analysis for AKD according to baseline prognostic factors after excluding patients with CKD.

| **Parameter** | **Beta**  **Coefficient** | **Standard error** | **Odds ratio (95% CI)** | ***p*-value** |
| --- | --- | --- | --- | --- |
| **Univariable logistic regression** | | | | |
| Age | 0.027 | 0.010 | 1.028(1.007-1.049) | 0.008 |
| Hemoglobin | -0.296 | 0.070 | 0.744(0.649-0.853) | <0.001 |
| Body temperature | 0.319 | 0.167 | 1.376(0.992-1.909) | 0.056 |
| Respiratory rate | 0.063 | 0.024 | 1.065(1.015-1.117) | 0.010 |
| Serum creatinine 0-24 h | 0.575 | 0.351 | 1.778(0.893-3.538) | 0.101 |
| Serum creatinine 24-48 h | 2.338 | 0.932 | 10.363(1.668-64.396) | 0.012 |
| Serum creatinine 48-72 h | 0.680 | 0.362 | 1.974(0.972-4.009) | 0.060 |
| hsCRP | 0.014 | 0.005 | 1.014(1.005-1.024) | 0.002 |
| Ejection fraction | -0.022 | 0.009 | 0.978(0.961-0.995) | 0.011 |
| BNP | 0.001 | 0.000 | 1.001(1.000-1.002) | 0.001 |
| Albumin | -1.349 | 0.370 | 0.260(0.126-0.536) | 0.001 |
| Serum IL-18 | 0.003 | 0.001 | 1.003(1.001-1.004) | 0.001 |
| Serum NGAL | 0.001 | 0.001 | 1.001(0.999-1.004) | 0.264 |
| Urine NGAL | 0.004 | 0.003 | 1.004(0.999-1.009) | 0.123 |
| Serum CysC | 0.000 | 0.000 | 1.000(1.000-1.001) | 0.067 |
| **Multivariable logistic regression** | | | | |
| Hemoglobin | -0.224 | 0.097 | 0.799(0.660-0.967) | 0.021 |
| Age | 0.026 | 0.014 | 1.027(0.998-1.056) | 0.064 |
| Ejection fraction | -0.028 | 0.012 | 0.973(0.951-0.995) | 0.017 |
| Serum IL-18 | 0.002 | 0.001 | 1.002(1.000-1.003) | 0.058 |

IL-18, interleukin 18; NGAL, neutrophil gelatinase-associated lipocalin; CysC, cystatin C; hsCRP, high sensitivity C-reactive protein; BNP, B-type natriuretic peptide; CKD, chronic kidney disease
